# Stem Cells from Healthy and Tendinopathic Human Tendons: Morphology, Collagen and Cytokines Expression and Their Response to T3 Thyroid Hormone

**DOI:** 10.3390/cells11162545

**Published:** 2022-08-16

**Authors:** Maria Camilla Ciardulli, Pasqualina Scala, Valentina Giudice, Antonietta Santoro, Carmine Selleri, Francesco Oliva, Nicola Maffulli, Giovanna Della Porta

**Affiliations:** 1 Laboratory of Translational Medicine, Department of Medicine, Surgery and Dentistry, University of Salerno, Via S. Allende, 84081 Baronissi, Italy; 2 Hematology and Transplant Center, University Hospital “San Giovanni di Dio e Ruggi D’Aragona”, 84131 Salerno, Italy; 3 Centre for Sports and Exercise Medicine, Barts and The London School of Medicine and Dentistry, Mile End Hospital, Queen Mary University of London, 275 Bancroft Road, London E1 4DG, UK; 4 Interdepartment Centre BIONAM, University of Salerno, Via Giovanni Paolo I, 84084 Fisciano, Italy

**Keywords:** tendon stem/progenitor cells, T3 thyroid hormone, tendinopathy, type III collagen, cytokines

## Abstract

The aim of this study was to investigate the effect of triiodothyronine (T3) on tendon specific markers and cytokines expression of stem cells extracted from human tendons. Indeed, thyroid hormones have been reported to be protective factors, maintaining tendons’ homeostasis, whereas tendinopathy is believed to be related to a failed healing response. Healthy and tendinopathic human tendons were harvested to isolate tendon stem/progenitor cells (TSPCs). TSPCs obtained from pathological samples showed gene expression and morphological modifications at baseline in comparison with cells harvested from healthy tissues. When cells were maintained in a medium supplemented with T3 (10^−6^ M), only pathological populations showed a significant upregulation of tenogenic markers (*DCN*, *TNC*, *COL1A1*, *COL3A1*). Immunostaining revealed that healthy cells constantly released type I collagen, typical of tendon matrix, whereas pathological ones overexpressed and secreted type III collagen, typical of scarred and impaired tissue. Pathological cells also overexpressed pro- and anti-inflammatory cytokines, suggesting an impaired balance in the presence of T3, without STAT3 activation. Moreover, DKK-1 was significantly high in the culture medium of pathological cell cultures and was reversed by T3. This study opens perspectives on the complex biochemical alteration of cells from pathological tendons, which may lead to the chronic disease context with an impaired extracellular matrix.

## 1. Introduction

Tendinopathy is generically referred to as a clinical condition in and around tendons arising from overuse and is defined as a clinical syndrome characterized by pain, swelling, and impaired performance [1,2,3]. The etiology of tendinopathy remains unclear, and many causes have been hypothesized, including hypoxia, ischemic damage, oxidative stress, or matrix metalloproteinase (MMP) imbalance [4,5]. Moreover, functional alterations, biomechanical factors, aging, and metabolic disorders are predisposing factors increasing the risk of re-injury [1,2]. The process of tendinopathy involves collagen matrix and tenocytes [6,7]. In physiological conditions, collagen fibers are tightly parallelly bundled, while tendinopathic tissues show unequal and irregular crimping, loosening and increased collagen waviness, with high expression of type III reparative collagen [6,7,8,9]. Moreover, specific tenocytes’ shapes and gene expression relate to certain tendinopathic conditions [10]. In pathological tendons, tenocytes are abnormally plentiful in some areas with rounded nuclei, increased production of proteoglycan and proteins, and a chondroid morphology; other areas display rare tenocytes with small pyknotic nuclei [11]. Tendinopathic tissues exhibit changes in expression of genes involved in cell–cell and cell–matrix interactions, with both upregulation (MMP-7) and downregulation (MMP-3) of genes involved in extracellular matrix (ECM) modeling, and upregulation of genes interested in ECM synthesis (COL1A1, COL3A1) [12,13]. Moreover, pro-inflammatory cytokines can initiate the catabolic process after injuries, including tumor necrosis factor-α (TNF-α), interleukin(IL)-1 β, and interleukin-6 (IL-6), growth factors, such as transforming growth factor β (TGF-β) or epidermal growth factor (EGF) [1,14,15], and transcriptional factors, especially in the early phases of tendinopathies [16]. 

Effects of thyroid hormones on ECM biosynthesis already have been described [17]. Triiodothyronine (T3) is important in systemic metabolism and regulates several gene expressions involved in cells morphology, differentiation, and proliferation. Moreover, it coordinates tendon ECM synthesis and organization [18,19]. Indeed, in hyperthyroidism, an increased catabolism of soluble and insoluble collagen can be observed, whereas in hypothyroidism, a decreased rate of collagen catabolism is documented [18,20,21]. Thyroid hormones can exert non-genomic activities not related to their transcriptional factor functions because integrins can also bind T3 and activate mitogen-activated protein kinase (MAPK)/ extracellular signal-regulated kinases (ERK)1/2 signaling pathways favoring intracellular trafficking and protein phosphorylation, including signal transducer and activator of transcription (STAT) proteins [22]. During hypothyroidism, STAT5A/B is reduced in lactating rat mammary gland models, while the Janus kinase (JAK) 1 and 2/STAT3 pathway is already known to be involved in thyrotropin (TSH) signaling transduction [23,24]. However, JAK/STAT signaling activation is mainly regulated by a fine-tuned balanced between pro- and anti-inflammatory cytokines present in the microenvironment; impairment in reparative inflammatory cytokine responses is related to maladaptive tendon repair [25,26].

A specific cell population extracted from tendon, the tendon stem/progenitor cells (TSPCs), has been recently described and first defined as tendon-resident mesenchymal stem cells (MSCs) with self-renewal and multi-differentiation potentials. TSPCs play key roles in tendon development, homeostasis, and healing [27,28], and TGFβ drives TSPCs commitment [29]. Wnt/β-catenin signaling is another important pathway involved in tendon healing, as activation of this pathway suppresses the expression of scleraxis (Scx) or tenomodulin (Tnmd) in tendon-derived rat cells [30]. TSPCs express tendon-related markers in vitro and can form tendon and enthesis-like tissues when implanted in vivo [31]. Therefore, TSPCs are an attractive alternative treatment for tendon diseases that often fail to yield a satisfactory outcome. Extraction and investigation of TSPCs in healthy and pathological conditions could unveil the peculiar characteristics of tendon tissues. Better understanding of their involvement in the reparative process could promote optimized manipulation of TSPCs and hasten progress of future clinical applications. 

Following these consideration, healthy (semitendinosus tendons) and tendinopathic (Achilles tendons) tissues were harvested from patients undergoing surgery, and in vitro TSPC cultures were established. Cell populations were identified by flow cytometry, and their morphology was investigated by microscopy. In a previous paper, the authors studied the effect of thyroid hormones on tenocytes harvested from healthy tendon biopsy, identifying 10^−6^ M as the best concentration for the treatment [18]. In the present work, a medium supplemented with T3 thyroid hormone was used to study the response of both healthy and pathological TSPCs, comparing their tenogenic and cytokine gene expression, as well as collagen deposition, to understand if T3 supplemented medium can have an influence on these expressions. Type I and III collagen, scleraxis (SCX-A), decorin (DCN) and tenascin-C (TNC) genes were monitored by quantitative real-time PCR (qRT-PCR), and the effect on type I and III collagen secretion along 14 days of culture was evaluated by immunofluorescence (IF). Pro- (IL-6, TNF, IL-12A and IL-1β) and anti-inflammatory (IL-10 and TGF-β1) cytokine expression was investigated by gene expression and immunoassay to study the involvement of inflammatory pathways in healthy and tendon healing with and without T3. STAT3 phosphorylation was also studied by flow cytometry to observe if inflammatory pathways in pathological cell populations was reversed by T3. 

## 2. Materials and Methods

A total of four healthy semitendinosus and four tendinopathic tendons were obtained after informed consent according to protocols approved by the Institutional Review Board of “San Giovanni di Dio e Ruggi D’Aragona Hospital” (Salerno, Italy) (Review Board prot./SCCE n. 151 achieved on 29 October 2020). Healthy tendon samples were collected from non-suitable tissue parts for semitendinosus autologous transplants after reconstruction of anterior cruciate ligament, whereas tendinopathic tendon samples were harvested from patients undergoing surgery following Achilles tendon traumas. In particular, one semitendinosus and one tendinopathic tendon were both harvested from one male patient (60 years old) following autologous reconstruction of Achilles tendon. Three healthy semitendinosus tendons were from three different subjects (males; 42, 55, and 37 years old), and the tendinopathic tendons were from other three separate patients (males; 53, 61, and 57 years old). Tendon composition should not greatly differ based on anatomical site as far as tendons and ligaments are not mixed [32]. Presence of comorbidities and any previous or concomitant anterior cruciate ligament/Achilles tendon disease were considered as exclusion criteria.

### 2.1. TSPCs Extraction 

Tendon samples were washed several times with sterile phosphate buffer saline (PBS) 1x (Corning Cellgro, Manassas, VA, USA), supplemented with 1% penicillin/streptomycin (Corning), and 1% amphotericin B (Corning); any visible fat, muscle, or paratenon were removed. Tendons were cut into small pieces, mechanically disaggregated with the aid of a scalpel, and placed in standard Petri dishes. A total of 3 mL of trypsin-ethylenediaminetetraacetic acid (EDTA) solution (Corning) was added to each dish and incubated for 30 min at 37 °C. Then, 12 mL of α-MEM (Corning) supplemented with 1% Glutagro^TM^ (Corning), 20% FBS (Gibco^TM^, Walthan, MA, USA), 1% penicillin/streptomycin (Corning, Manassas, VA, USA), and 1% amphotericin B (Corning) were used to neutralize trypsin activity. The cells present were left to adhere at 37 °C with 5% CO_2_ and air in α-MEM medium (supplemented as described above) with a change every 2–3 days. Once cell confluency reached 70–80%, cells that migrated from tendon pieces were detached using 0.25% trypsin–2.21 mM EDTA in 1X solution and centrifuged at 1400 rpm for 10 min. The phenotype of collected cells was analyzed by flow cytometry and used for culture at passage 1 to avoid phenotype drift. 

### 2.2. Flow Cytometry and Gating Strategy

Cells were detached and counted, and 1 × 10^5^ cells were incubated at room temperature (RT) for 20 min with the following directly conjugated mouse–anti-human antibodies: CD34-PE, CD90-FITC, CD105-PE, CD45-PC7, HLA class-II-FITC, and CD14-PC7 (all from Beckman Coulter, Fullerton, CA, USA), and CD73-APC (Miltenyi Biotec, Gladbach, Germany). After antibody incubation, samples were washed twice with PBS 1x (Corning Cellgro) and resuspended in the same buffer for acquisition. For phosphoprotein analysis (phosFlow), pSTAT3-PerCP-Cy5.5 and pSTAT5-Alexa Fluor 488 were used as previously reported [33]. Samples were acquired with a BD FACSVerse flow cytometer (Becton Dickinson, BD, Franklin Lakes, NJ, USA), equipped with 2 lasers (blue: 488 nm, and red laser: 628 nm). Compensation was calculated using single-color controls for each fluorochrome and an unstained sample as negative control for setting PMT voltages. All samples were run using the same PMT voltages. A minimum of 30,000 events were recorded. FlowJo software (v.10.7.1, LLC, BD Biosciences, Franklin Lakes, NJ, USA) was employed for post-acquisition compensation and flow cytometric analysis. TSPCs were first identified using linear parameters (forward scatter [FSC] area [FSC-A] vs. side scatter area [SSC-A], and double cells were excluded (FSC-A vs. FSC-H). Expression of each marker on single cells was reported using histograms and an unstained sample as negative control. For phosFlow analysis on single cells, CD73^+^ TSPCs were identified, and pSTAT3 expression was investigated. Results were reported as median fluorescence intensity (MFI), and fold change was calculated as the ratio between T3-stimulated and unstimulated (ctr) samples. 

### 2.3. T3 Treatment

TSPCs were seeded at 1 × 10^4^ per well in a 12-well plate and cultured in α-MEM (Corning) supplemented with 1% Glutagro^TM^ (Corning), 10% fetal bovine serum (FBS) (Gibco^TM^), and 1% penicillin/streptomycin (Corning). After 48 h, cells were exposed to T3 (10^−6^ M) and L-ascorbic acid (10^−7^ M), as previously optimized [18]. Treatment was repeated every 3 days, concurrently with medium change. At days 1, 7, and 14, cells were collected for qRT-PCR and immunofluorescence (IF) assays.

### 2.4. Brightfield Images

Brightfield images were captured at different time points at 5× magnification (zoomed area: 150%) using a Leica DMIL LED microscope and acquired by Leica DFC425 C Camera. 

### 2.5. Total RNA Isolation and Gene Expression by qRT-PCR

Total RNA was extracted from TSPCs using the RNeasy Mini Kit (Qiagen, Hilden, Germany). Then, the iScript^TM^ cDNA synthesis kit (Bio-Rad, Milan, Italy) was used to reverse-transcribe 1 μg of total RNA for each sample. Relative gene expression analysis was performed in a LightCycler^®^ 480 Instrument (Roche, Monza, Italy), using the SsoAdvanced^TM^ Universal SYBR^®^ Green Supermix (Bio-Rad) and the validated primers for SCX-A, DCN, TNC, COL1A1, COL3A1, IL-6, TNF, IL-12A, IL-1β, IL-10, and TGF-β1 (Bio-Rad), according to MIQE guidelines [34]. Triplicate experiments were performed for each condition studied, and data was normalized to *GAPDH* expression. The geNorm method [35] was applied to calculate reference gene stability between the different conditions (calculated with CFX Manager software [v.3.1; Bio-Rad, Milan, Italy]; M < 0.5). Fold changes were determined using the 2^−ΔΔCt^ method and presented as relative levels over T0 = 1.

### 2.6. Immunofluorescence Assay

Cells were fixed with 3.7% formaldehyde for 30 min at RT followed by permeabilization with 0.1% Triton X-100 for 5 min and blocking with 1% bovine serum albumin (BSA) for 1 h. 

For type I and type III collagen staining, cells were incubated overnight at 4 °C with a rabbit polyclonal anti-type I collagen antibody (1:200, Abcam, Cambridge, UK) and a mouse monoclonal anti-type III collagen antibody (1:100; Santa Cruz Biotechnology, Dallas, TX, USA). Following incubation with the primary antibody, cells were incubated for 1 h at RT with the Alexa Fluor^TM^ 488 goat–anti-rabbit IgG (1:500; Thermo Fisher Scientific, Waltham, MA, USA) and the Alexa Fluor^TM^ plus 594 goat–anti-mouse IgG (1:500; Thermo Fisher Scientific) antibodies. For β-actin staining, cells were incubated for 1h at RT with a mouse monoclonal anti-β actin antibody (1:100, Cell Signaling Technology, Danvers, MA, USA) and then for 1 h at RT with the Alexa Fluor^TM^ plus 594 goat–anti-mouse IgG (1:500; Thermo Fisher Scientific) antibody.

Cell nuclei were stained with 4′,6-diamidino-2-phenylindole (DAPI) solution (1:1000) for 5 min. All images were acquired at 20× magnification (zoomed area: 150%) with identical setting of light, exposure time, and gain using a fluorescence microscope (Eclipse Ti Nikon Corporation, Tokyo, Japan).

### 2.7. Immunohistochemical Assay

Healthy and tendinopathic tissue samples were fixed in 3.7% formaldehyde (4 °C, overnight), cryo-protected in 30% sucrose overnight, mounted in OCT embedding compound, frozen at −20 °C, and then cut in slices of 8 μm of thickness using a cryostat. Slices were permeabilized with 0.1% Triton X-100 for 15 min, and nonspecific staining blocked with 1% BSA for 1 h at RT. For type I and type III collagen staining, slices were incubated overnight at 4 °C with a mouse monoclonal anti-type I collagen antibody (1:100, Sigma Aldrich, Milan, Italy) and a mouse monoclonal anti-type III collagen antibody (1:200, Sigma Aldrich). Following incubation with the primary antibody, slices were incubated for 1 h at RT with the DyLight 488 goat–anti-mouse IgG (1:500, BioLegend, San Diego, CA, USA) and the Alexa Fluor^TM^ plus 594 goat–anti-mouse IgG (1:500; Thermo Fisher Scientific) antibodies, respectively. Subsequently, cell nuclei were stained with DAPI solution (1:1000) and incubated for 10 min. Image acquisition was at 20× magnification on a fluorescence microscope (Eclipse Ti Nikon Corporation, Tokyo, Japan), using identical setting of light, exposure time, and gain.

### 2.8. Multiplex Immunoassay

A bead-based multiplex custom immunoassay (9-plex LEGENDplex^TM^ Custom Panel; BioLegend, San Diego, CA, USA) was employed for measurement of IL-1β, IL-6, TNF-α, hepatocyte growth factor (HGF), IL-15, IL-10, macrophage inflammatory protein (MIP)-1α and 1β, and Dickkopf-related protein 1 (DKK1) in supernatants of culture media diluted 1:50 with fresh complete α-MEM. Samples were run in duplicate, and cytokine quantification was performed by converting median fluorescence intensity to concentrations using a calibration made with standards as per manufacturer’s instructions. A total of 3400 total beads (1200 Beads A and 1200 Beads B) were recorded using a BD FACSVerse cytometer (BD Biosciences, NJ, USA) equipped with a blue (488 nm) and a red laser (628 nm) and BD FACSuite software (BD Biosciences). Post-acquisition analysis was performed using LEGENDplex™ Data Analysis Software Suite (BioLegend).

### 2.9. Statistical Analysis 

Statistical analysis was performed using GraphPad Prism software (v6.0 for Windows, LLC, San Diego, CA, USA). Data obtained from multiple experiments (n = 3) are calculated as mean +/− SD and analyzed for statistical significance using ordinary one-way analysis of variance (ANOVA) test, for independent groups. Differences were considered statistically significant when *p* < 0.05.

## 3. Results

### 3.1. TSPCs Isolation and Characterization from Healthy and Pathological Tendons

**Figure 1:**
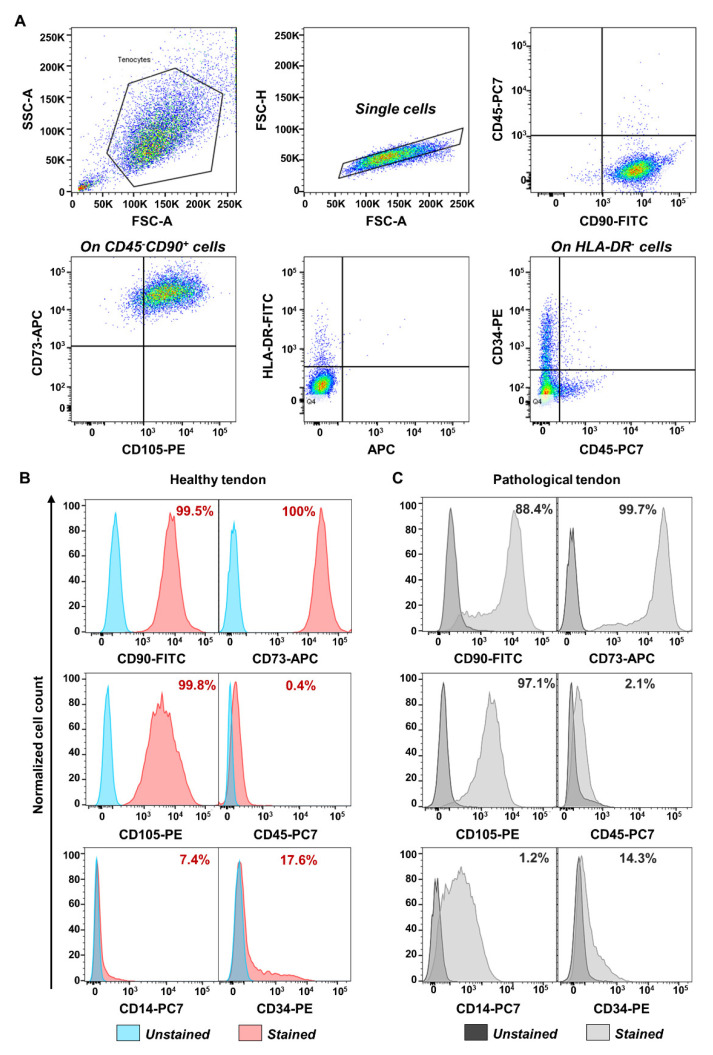
Flow cytometry characterization of tendon stem/progenitor cells (TSPCs) extracted from healthy and pathological tendon samples. (**A**) The panel shows representative flow cytometry events on forward scatter (FSC) vs. side scatter (SSC), excluding double cells (FSC-A vs. FSC-H) and further studying CD45, CD90, CD73, CD105, HLA-DR, CD34, and CD14 surface marker expression. Normalized cell count histograms display marker expression on single cells in (**B**) healthy and (**C**) pathological tendon.

**Figure 2:**
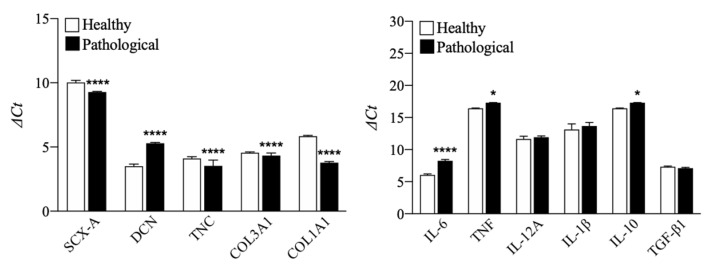
qRT-PCR characterization of tendon stem/progenitor cells (TSPCs) extracted from healthy and pathological tendon samples. The histograms show gene expression profile for tenogenic (SCX-A, DCN, TNC, COL3A1, COL1A1) and cytokines (pro-inflammatory: IL-6, TNF, IL-12A, IL-1β; anti-inflammatory: IL-10, TGF-β1) markers before treatment (day 0). Data were expressed as ΔCt, normalizing each gene of interest to GAPDH (housekeeping gene). Results are shown as mean ±SD. * *p* < 0.05 and **** *p* < 0.0001 (N = 4).

TSPCs were extracted, harvested, and characterized by flow cytometry. The analysis indicated that both healthy and pathological tissue-derived TSPCs expressed positive mesenchymal stem cells surface markers (CD90, CD73) and CD105 (a TGF-β receptor accessory molecule), but negative for CD45, HLA-DR, CD34, and CD14, except for pathological TSPCs that showed positivity for CD14 [36] (see Figure 1). Moreover, at day 0, before treatment, compared to healthy TSPCs, pathological TSPCs showed slight but significant (*p* < 0.0001) downregulation of tendon specific markers, except for DCN (a component of tendon ECM) that was upregulated (*p* < 0.0001). However, we found overexpression of some pro-inflammatory cytokines, such as IL-6 (*p* < 0.0001) and TNF (*p* < 0.05), in pathological cells, compared to healthy ones (see Figure 2), suggesting that TSPCs can regulate inflammation during tendon healing [37].

**Figure 3:**
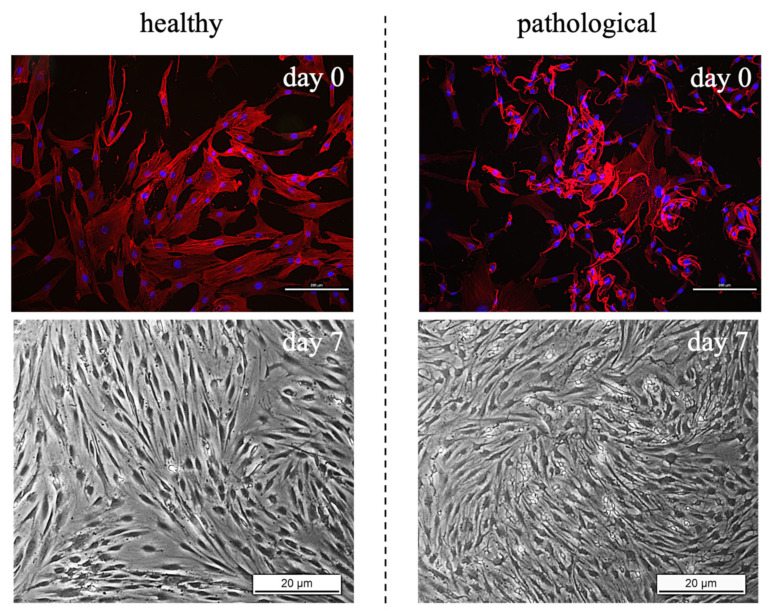

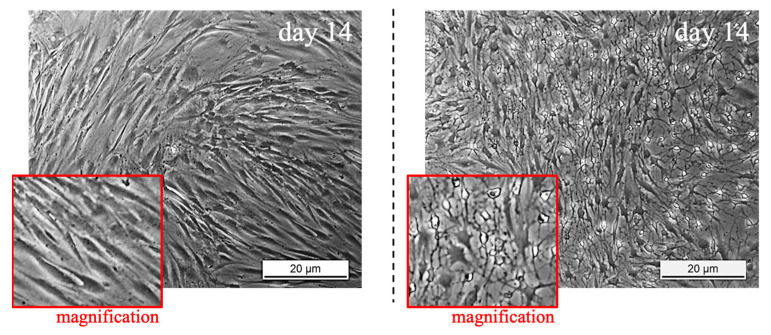
Brightfield and immunofluorescence images illustrating the shape of tendon stem/progenitor cells (TSPCs). Cells extracted from healthy tendons showed specific alignment and elongated shape modification (fibroblast-like), whereas cells obtained from pathological tendons exhibited a large perinuclear area with thin dendrites (zoomed area: 150%). β-actin staining (red) was used to display cells cytoskeleton. Scale bar: 20 μm.

TSPCs morphological investigation indicated a different shape between healthy and pathological tissue-derived TSPCs, as observed in Figure 3. Indeed, cells extracted from healthy tendons showed specific alignment and elongated shape modification (fibroblast-like), whereas cells obtained from pathological tendons exhibited a smaller cell body with thin dendrites in the perinuclear area, especially after 14 days of culture. However, the different morphology between healthy and pathological tissue-derived TSPCs was already evident at day 0, when β-actin staining was used to visualize cells’ cytoskeleton.

**Figure 4:**
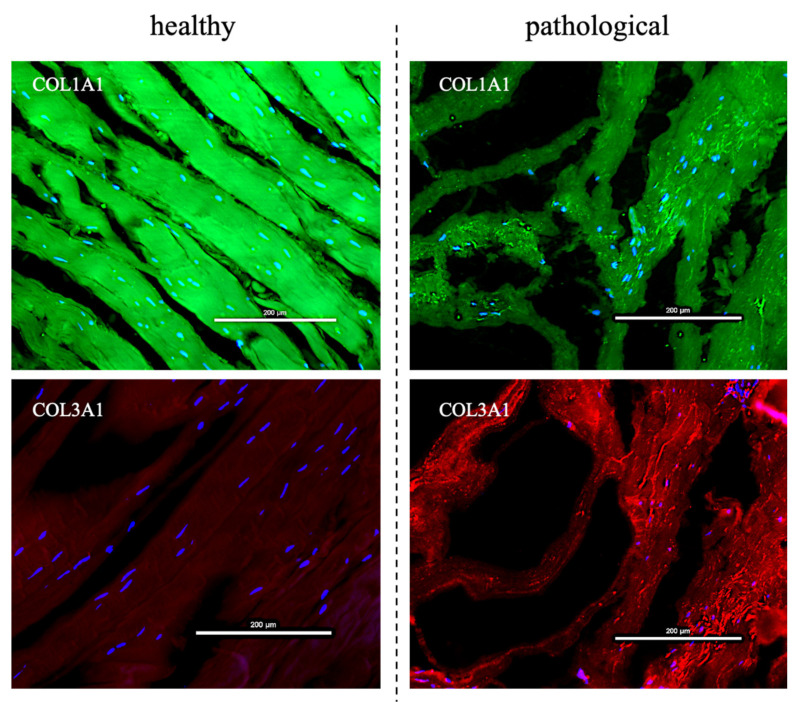
Immunofluorescence images of type I (green) and type III (red) collagen expression in healthy and pathological tendon samples. Images show that the healthy tendon has well shaped and organized fibers with a prevalence of type I collagen, whereas the pathological tissue has less organized, randomly oriented fibers and with a heavy presence of type III collagen. Scale bar: 200 μm.

Considering that pathological cells showed a different morphology, healthy and tendinopathic tissue samples also were observed highlighting both type I and III collagen proteins by IF. The tissue samples showed again a different extracellular matrix organization also with a different collagen composition. Healthy tendons showed well shaped and organized fibers with a prevalence of type I collagen, whereas the pathological tissue exhibited less organized and randomly oriented fibers, with a heavy presence of type III collagen (see Figure 4).

### 3.2. Evaluation of T3 Hormone Effect on Collagen Production

**Figure 5:**
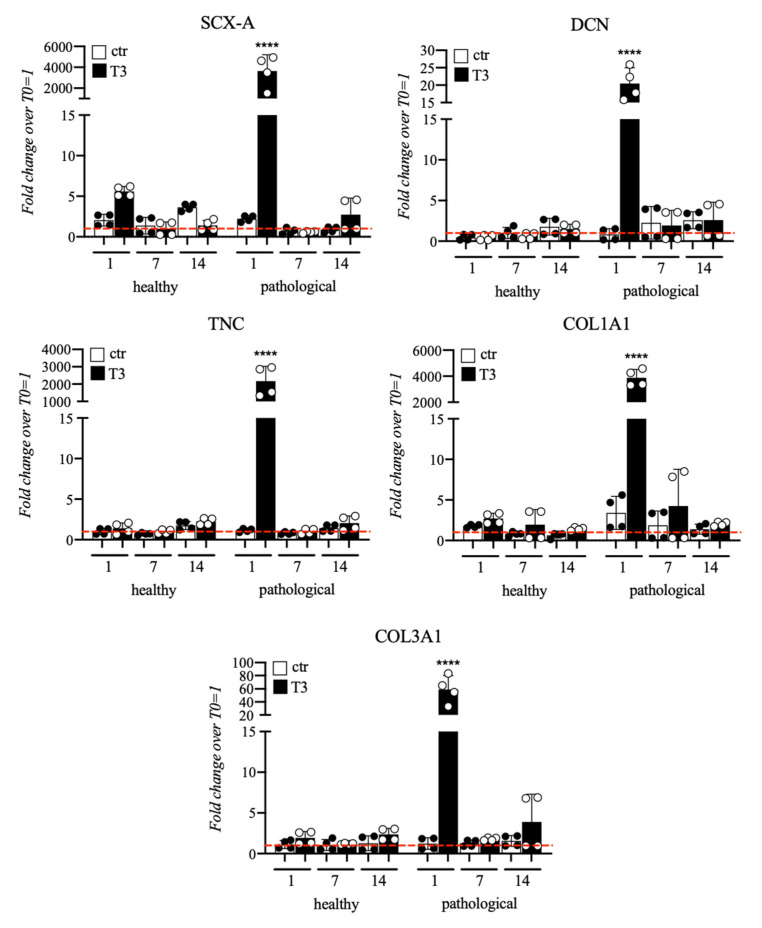
Gene expression profile for tenogenic markers of tendon stem/progenitor cells (TSPCs) cultured in a medium supplemented with T3 (10^−6^ M) up to 14 days. Healthy cells showed a rather constant mRNA expression of different tenogenic markers, whereas cells extracted from pathological tendons exhibited larger overexpression of SCX-A, DCN, TNC, COL1A1, and COL3A1 at Day 1. Results are shown as mean ± SD. **** *p* < 0.0001 (N = 4).

Healthy and tendinopathic tissue-derived tendon stem/progenitor cells (TSPCs) were cultured in a medium supplemented with T3 for 14 days to ascertain if the presence of the hormone can improve their collagen production or change tendon specific markers expression. The T3 concentration was of 10^−6^ M, as previously optimized [18]. Cells were characterized not only by qRT-PCR exploring the main tenogenic gene expression, such as type I and type III collagen but also by SCX-A, DCN and TNC, as reported in Figure 5.

**Figure 6:**
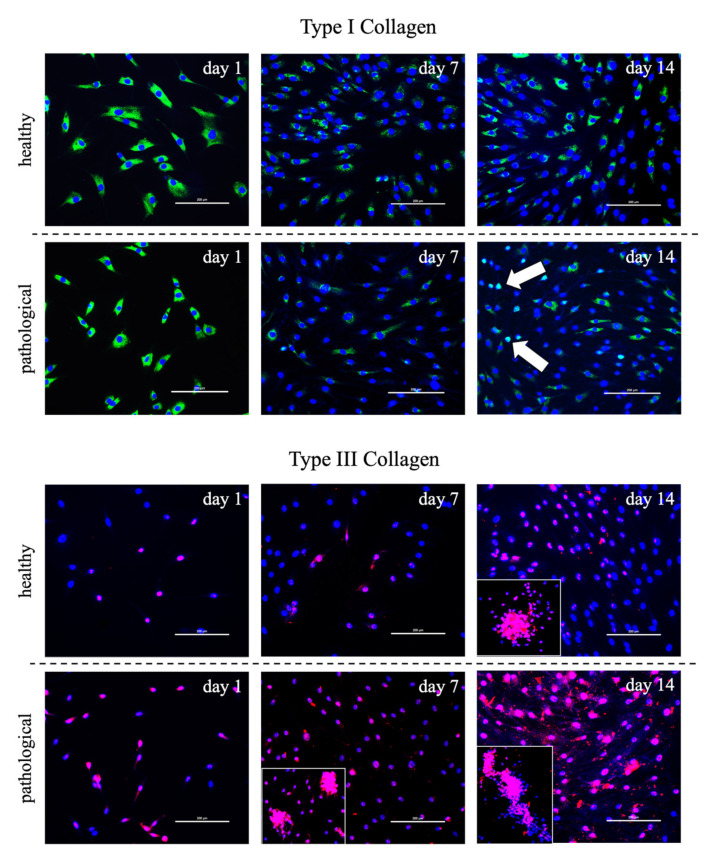
Immunofluorescence images of healthy and pathological tissue-derived tendon stem/progenitor cells (TSPCs) cultured in a medium supplemented with T3 (10^−6^ M) up to 14 days. Type I (green) and III (red) collagen expression was monitored. DAPI was used to counterstain the nuclei (blue). Pathological cells exhibited an ovoid morphology (white arrow) and tended to form aggregates with enhanced type III collagen secretion (zoomed area: 150%). Scale bar: 200 µm.

TSPCs extracted from healthy tendon samples showed a constant expression of tenogenic markers, without significant variations over culture time. Instead, significant up-regulation of the main markers analyzed was observed in pathological tissue-derived TSPCs treated with T3. *SCX-A* expression appeared significantly upregulated at Day 1 (4000-fold) in T3-treated TSPCs from pathological tendon samples compared to untreated cells. *DCN* showed again a higher upregulation in tendinopathic tissue-derived TSPCs (20-fold at Day 1) compared to TSPCs extracted from healthy tendons. Similarly, *TNC* was significantly overexpressed in pathological cells only at Day 1 (2000-fold). At the same time point, pathological tissue-derived TSPCs retained a strong increase in *COL1A1* (4000-fold) and *COL3A1* (60-fold). In addition to gene expression difference between healthy and pathological cell populations, morphological differences were observed by immunofluorescence assay for type I and III collagen along the culture, as illustrated in Figure 6. Particularly, pathological cells always exhibited an ovoid morphology and formed aggregates, and mainly secreted type III collagen, whereas healthy cells showed a fibroblast-like shape and constantly secreted type I collagen.

### 3.3. Evaluation of T3 Hormone Effect on Cytokines Expression

**Figure 7:**
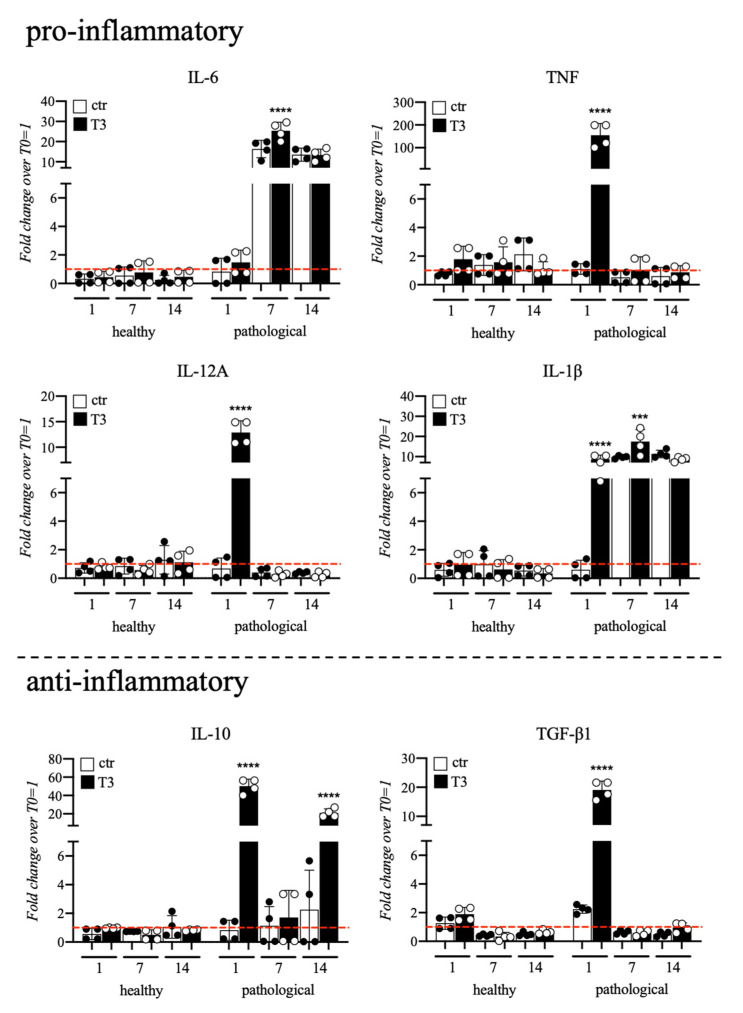
Gene expression profile for pro-/anti-inflammatory cytokines of tendon stem/progenitor cells (TSPCs) cultured in a medium supplemented with T3 (10^−6^ M) up to 14 days. Compared to healthy cells, the ones extracted from pathological tendons showed an impaired cytokines balance. Results are shown as mean ± SD. *** *p* < 0.001 and **** *p* < 0.0001 (N = 4).

Cytokines gene expression profile of healthy and pathological TSPCs cultured in a medium supplemented with T3 up to 14 days was also monitored, and data are summarized in Figure 7. Compared to healthy, pathological tendons, cells showed upregulation of pro-inflammatory cytokines with significant overexpression of *TNF* (180-fold), *IL-12A* (13-fold), and IL-1β (10-fold) at Day 7; upregulation of IL-6 (25-fold) and IL-1β (18-fold) was noted at Day 7. Pathological cells also showed significant overexpression of anti-inflammatory ones, such as IL-10 (50-fold at Day 1 and 35-fold at Day 14) and TGF-β1 (20-fold) at Day 1 of culture. Conversely, healthy cells did not show any significant event of cytokines overexpression.

**Figure 8:**
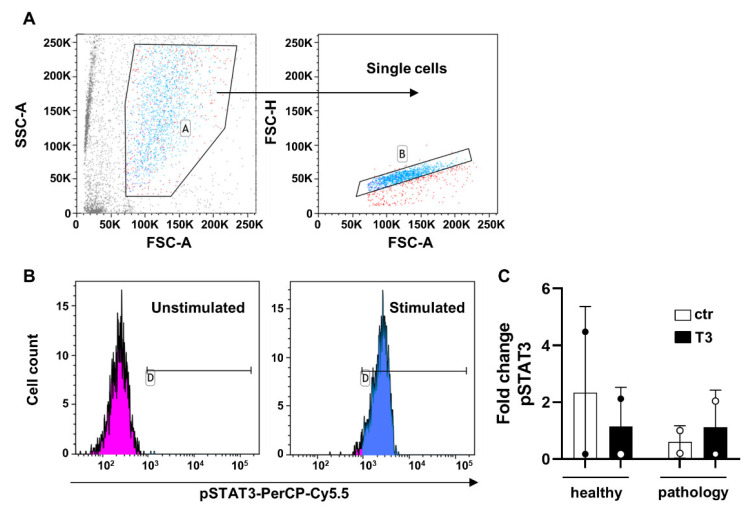
Phosphorylation status analysis of STAT3 by flow cytometry. Healthy and pathological TSPCs were treated (T3) or not (ctr) with T3 for 20 min and then subjected to fixation, permeabilization, antibody staining, and acquisition using a BD FACSVerse flow cytometer. (**A**) TSPCs were identified using linear parameters first (forward side scatter [FSC]-area vs. side scatter-area [SSC-A]; gate A), and then double cells were excluded (FSC-A vs. FSC-H; gate B). On single cells, (**B**) pSTAT3 expression was investigated. Data are shown as cell count histograms, and results are reported as median fluorescence intensity (MFI) or percent of positive cells (gate D). (**C**) Fold change variations of pSTAT3 expression was expressed as the ratio between T3-stimulated and unstimulated samples (N = 2). Results are shown as mean ± SD.

PhosFlow analysis was performed for investigation of changes in STAT3 phosphorylation status after T3 stimulation; in the experiments, we observed that T3 stimulation did not influence STAT3 signaling in both healthy and disease-derived TSPCs, as summarized in Figure 8.

**Figure 9:**
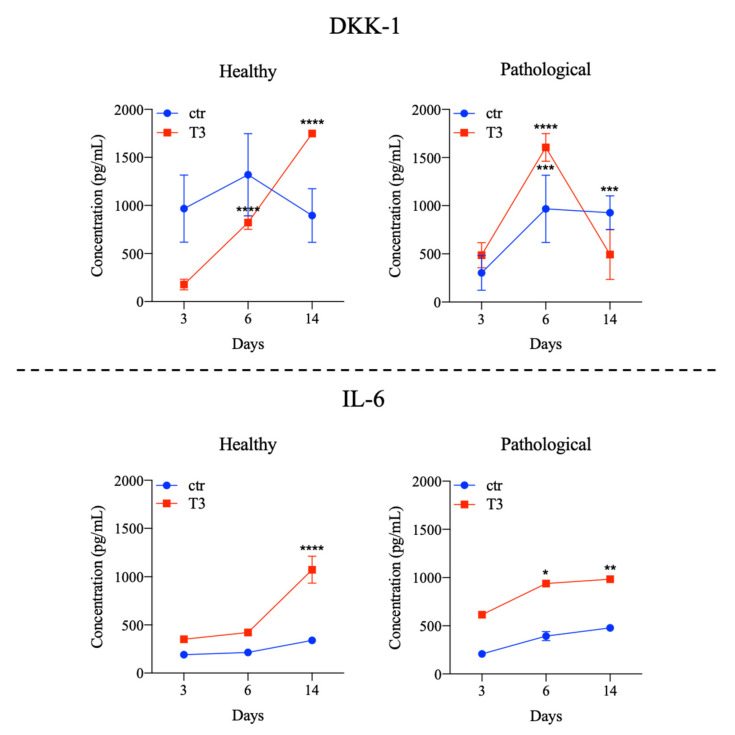
DKK-1 and IL-6 profiles of healthy and pathological tissue-derived tendon stem/progenitor cells (TSPCs) cultured in a medium supplemented with T3 (10^−6^ M) up to 14 days. Cytokine levels (pg/mL) were measured in culture medium at various time points (3, 6, and 14 days) using a bead-based multiplex immunoassay. Results are shown as mean ± SD. * *p* < 0.05, ** *p* < 0.01, *** *p* < 0.001, and **** *p* < 0.0001 (N = 2).

Cytokine levels were also measured by immunoassay, as shown in Figure 9 and Figure S1. In supernatants of healthy tissue-derived TSPCs treated with T3, DKK1 levels were significantly increased at Days 6 and 14, while IL-6 only at Day 14. Pathological TSPCs showed higher production of DKK-1 at Days 6 and 14 even when cultured in standard medium, dropping at Day 14 in the presence of T3. IL-6 remained significantly increased from Days 6 to 14 (Figure 9). For other cytokines explored, we did not find any significant event of overexpression either in healthy or pathological cells (see Figure S1 of Supplementary Materials).

## 4. Discussion

Response to tendon injury goes through three overlapping stages. In the inflammatory stage, during the first few days at the wound site, a fibrin clot is formed to provide temporary stiffness allowing macrophages to digest necrotic debris. Then, tenocytes are recruited and start to proliferate, particularly in the epitenon. In the second stage, termed proliferative or repair stage, an intense synthetic activity is triggered by macrophages and tenocytes. Macrophages release growth factors and direct cell recruitment; meanwhile, tenocytes can deposit a temporary, mechanically inferior matrix formed mostly of type III collagen. In the third and final stage, the remodeling phase, type I collagen synthesis prevails, and ECM is reshaped and aligned. This last phase begins 1–2 months after injury and can last more than a year. The repaired tissue often appears scar-like and does not completely regain its biomechanical properties [4,38,39].

Despite the large abundance of ECM in tendon, very little is known about its role in regulating the function of the cells that reside within it. In 2007, Bi et al. showed, for the first time, that human and mouse tendons harbor a unique cell population, termed tendon stem/progenitor cells (TSPCs), that has universal stem cell characteristics, such as clonogenicity (the ability to form clones), multipotency (multi-differentiation potential toward osteogenesis, adipogenesis, and chondrogenesis), and self-renewal capacity (higher doubling capacity than bone marrow stromal cells from the same sources). The isolated TSPCs could regenerate tendon-like tissues after extended expansion in vitro and transplantation in vivo [27]. However, the understanding of how stem/progenitor cells contribute to tendon healing and pathology is still quite limited, particularly regarding inflammation and matrix remodeling processes. For example, it has been recently showed that TSPCs regulate inflammation during tendon healing [37]; furthermore, they commit toward the expected cell lineages in situ or secrete chemotactic molecules/growth factors, which can recruit extra reparative cells into the lesion site [40,41].

We extracted and characterized TSPCs from both healthy and pathological tendon samples. Surgeries were always performed within 3–5 days after traumas, and TSPCs were isolated immediately; therefore, we presumably were in the initial part of the tendon healing process. Flow cytometry characterization of extracted TSPCs showed that these cells conserved a MSC phenotype with positivity for CD90, CD73, and CD105, and negativity for CD45, HLA-DR, and CD34, as defined by the International Society of Cellular Therapy [42].

Little is known about the potential phenotype drift of TSPCs after an injury, but it has been clearly demonstrated that damaged tendons are characterized by tenocytes phenotypic changes, with a more fibrocartilaginous matrix [43]; in fact, we noticed from morphological investigations that TSPCs isolated from pathological tissue samples were more rounded and are reminiscent of chondrocytes compared to TSPCs extracted from healthy tendons, which exhibited a clear fibroblast-like shape. 

The described pathological cell shape suggested an impaired metabolism, which may lead to an impaired extracellular matrix ECM. Indeed, tendon ECM is mainly formed by multilevel organized collagen. This protein forms fiber-like structures at a number of different hierarchical levels, each aligned closely to the long axis of the tissue (the loading direction), conferring excellent uniaxial mechanical strength to the tendon [44]. A prevalence of organized fibers of type I collagen was found in healthy tendons when compared to tendinopathic tissue samples, which mainly expressed type III collagen. Type I collagen is the most abundant into the tendon matrix. It is the major component of tendon tissue (75–85% of the dry mass of tendon) and is responsible for its mechanical strength. Type III collagen, on the contrary, has a role in tendon healing. It is mainly responsible for fibrotic and scarred tissue arrangement and has been consistently reported at the rupture site of human tendons. The presence of this collagen type is thought to cause weakening in the tendon tissue, since its fibers are thinner and more extensive than those of type I [45,46]. 

The role of thyroid hormones (THs), T3 and thyroxine (T4), in the development and metabolism of many tissues and organs, both in early and adult life, is mainly mediated through T3, which regulates gene expression by binding to the TH receptors (TR)-α and -β [47,48,49,50]. Thyroid hormones have been reported to modulate cells morphology, differentiation, and proliferation, and to regulate ECM organization and synthesis also in tendon tissue [17,18,51]. Epidemiology investigation indicated that patients with thyroid disorders have a higher probability to develop tendinopathy [21]. The normal T3 concentration in human serum approximates 2 ng/mL [52,53,54], while in hyperthyroidism T3 concentration in serum is about 7 ng/mL [52]. The hormone concentration used in the present manuscript (0.6 μg/mL) could be high for serum. However, authors in a previous paper reported a clinical application of T3 in terms of a local injection in the injured site [55]. In that study, the authors administered T3 (0.1 μg/mL) by local injection directly in tendon defects; this concentration achieved interesting results in terms of healing the Achilles tendon injury in rat models.

Previous studies on T3 and tendon regeneration has shown that thyroid hormone can produce a better healing of tendon injury in animal models [55]. Moreover, T3 and T4 play an antiapoptotic role on tenocytes and can influence extra cellular matrix proteins secretion in vitro, enhancing collagen production [17]. However, to our knowledge, no data on the response of resident healthy and/or pathological TSPCs to thyroid hormones have been already reported.

Following these indications, we treated healthy and tendinopathic tissue-derived TSPCs with T3 at 10^−6^ M for 14 days, to verify the ability of the hormone to improve cells Collagen production (type I and type III collagen), along with changes in tendon specific gene expression (SCX-A, DCN, TNC).

SCX-A is known as a neotendon marker, expressed in pro-tendon sites in the developing embryo. Specifically, SCX-A is a tendon-specific basic helix-loop-helix transcription factor, which regulates tendon formation and several other characteristic genes, such as type I collagen and decorin. SCX transduction can lead to the direct commitment of MSCs into tendon progenitors [56], enhancing repair when clinically translated, in vivo, in tendon injury models [57], suggesting its involvement in the tendon’s attempt to repair damages in response to external stimuli. This deduction was confirmed by our experiments, where SCX-A was found to be strongly and significantly upregulated at Day 1 of treatment in TSPCs extracted from pathological tendon samples.

DCN, a small leucine-rich proteoglycan implicated in fibrillogenesis regulation, is a fundamental component of the tendon ECM. Increased deposition of DCN was found in injured tendons, compared to normal ones [58,59]. This evidence was confirmed in our experiments and even emphasized. Moreover, an alteration in TNC expression is reported to be associated with human tendon degeneration. TNC is generally associated with organized, fibrous regions of the tendon matrix that are typical of the normal tendon structure. This distribution is consistent with a role for TNC in collagen fibrils organization, perhaps maintaining the interface between fibrils and adjacent structures. Second, although TNC is generally absent in a poorly organized matrix of degenerate tendons, it is strongly associated with some rounded cells in disorganized fibrocartilaginous regions that are more abundant in pathological specimens [60]. This indication is consistent with the gene expression observed in our pathological cell samples. 

The study suggested that morphological and gene expression variation can be observed between healthy and pathological cells and that pathological TSPCs enhanced type III collagen secretion, probably inducing an impaired extracellular matrix assessment, as confirmed by immunofluorescence assay. However, the presence of T3 hormone promoted an increase in collagen production in pathological cells, which should reasonably have specific receptors. However, the specific collagen type expressed depends on healthy or pathological conditions, with an increase in disorganized type III collagen deposition in pathological samples, without any specific action of switching to type I collagen production. Our results suggested that T3 treatment could trigger this “collagen switch”. It is very likely that the TSPCs morphology and responses to T3 were only transient due to the course of healing phases, but despite the growing body of recent studies on TSPCs, we did not find any indications in the literature to support this hypothesis because the evolution of TSPCs morphology during tendon healing stages has never been described.

Given the key role of inflammation in tendon healing and repair, we also investigated the effect of T3 hormone on cytokines expression and release, which has been poorly investigated in previous studies. We found that pathological samples showed an overexpression of pro-inflammatory cytokines (IL-6, TNF, IL-12A, and IL-1β) and an overall impaired cytokines balance, which may be responsible for the secretion of the type III collagen protein, often associated with scar tissue that is frequent after an inflammation event [61,62,63]. In this context, the presence of T3 seemed a metabolic activator promoting the overexpression of an impaired cytokines path, without any reversing therapeutical activity of cytokines gene expression. Inflammation triggered by a tendon injury deeply impairs tenocyte gene expression, as documented by an upregulation of inflammasome-related genes, such as NLRP3 and Toll-like receptors 2 and 4 (TLR2/4), after rotator cuff tendon injuries, and inflammatory proteins resulting in ECM disorganization [64]. Moreover, during tendon injury and repair, a small fraction of circulating monocytes might differentiate in fibrocytes becoming positive for collagen I and CD34 while negative for CD14, a TLR4 co-receptor [65]. Conversely, a small population of TSPCs in healthy patellar tendons shows CD14 expression suggesting that tendon progenitors might lose or acquire this marker based on microenvironment stimulations [36]. Herein, we showed that pathological TSPCs also displayed a higher CD14 expression together with increased inflammatory cytokines compared to healthy tendon progenitors, suggesting that an aberrant response to inflammation could cause impaired tendon repair. 

Using JAK/STAT signaling pathway activation mainly regulated by a fine-tuned balance between pro- and anti-inflammatory cytokines, we also evaluated STAT3 phosphorylation after treatment, but it seemed not to be affected by T3 stimulation. This preliminary investigation confirmed an impaired cellular biochemistry of pathological cells that is responsible for the observed cellular morphology and the overall tendon matrix degeneration. The study of a complex biochemical pathway involved in the pathological events is not the aim of the present work, but these preliminary results open further questions and suggest that deeper biochemical investigation in pathological cells have to be performed to better explain all the adverse events that lead to a characteristic astrocyte-like cellular morphology and impaired extracellular matrix. Furthermore, the proposed results on JAK/STAT signaling involvement in tendinopathy are not conclusive and require further in vitro studies, also using well-known JAK inhibitors or agonists, to confirm the putative role of this pathway in normal and pathological conditions. 

Wnt/β-catenin signaling is also involved in tendon healing, as activation of this pathway can suppress the expression of Scx, or Tnmd in tendon cells [30], and DKK-1, a potent Wnt inhibitor [66], might positively influence tendon healing, as high levels were found in the supernatants of pathological TSPCs treated with T3, hypothetically leading to Wnt/β-catenin inactivation and subsequent Scx upregulation. Finally, data on cytokines expression might suggest a role of TSPCs in modulate immune responses and microenvironment composition. Further investigations are required to understand perturbations in immune cell composition and gene expression, especially in tendinopathies. 

## 5. Conclusions

The paper reported a successful TSPCs extraction strategy from tendon explants and demonstrated morphological and gene expression modifications for pathological TSPCs, in comparison with cells extracted from healthy tissues. Indeed, TSPCs showed different morphology in dependence on their healthy and pathological condition, probably due to impaired biochemical pathways, with overexpression of different collagen types, such as type III collagen, by pathological ones. Furthermore, a characteristic pro-inflammatory cytokines overexpression was detected in pathological cells.

T3 hormone might improve tenogenic marker expression by TSPCs likely acting as a metabolic promoter and as an inductor of Wnt/β-catenin inactivation, especially in pathological tissue-derived cells, while not influencing cytokine expression and production or downstream signaling. 

The study opens perspectives on the complex biochemical alteration of pathological TSPCs that could be important in monitoring chronic disease behavior and indicated that a better knowledge of their pathological and impaired biochemical pathways may also help in understanding a possibility to reverse these activities and promote healing events. However, our results suggested TSPCs are interesting cells for the development of in vitro models for tendon regeneration and healing.

## Data Availability

Not applicable.

## Supplementary Materials

The following supporting information can be downloaded at: https://www.mdpi.com/article/10.3390/cells11162545/s1, Figure S1: Cytokine profiles of healthy and pathological tissue-derived Tendon Stem/Progenitor Cells (TSPCs) cultured in a medium supplemented with T3 (10-6 M) up to 14 days.
