# Supplementary material for: Stem Cells from Healthy and Tendinopathic Human Tendons: Morphology, Collagen and Cytokines Expression and Their Response to T3 Thyroid Hormone
Source: Cells. 2022 Aug 16;11(16):2545. doi: 10.3390/cells11162545 (PMC9406581; doi:10.3390/cells11162545)
Supplement: Supplementary file 1 [file cells-11-02545-s001.zip › cells-1770281-supplementary.pdf]

Supplementary Materials

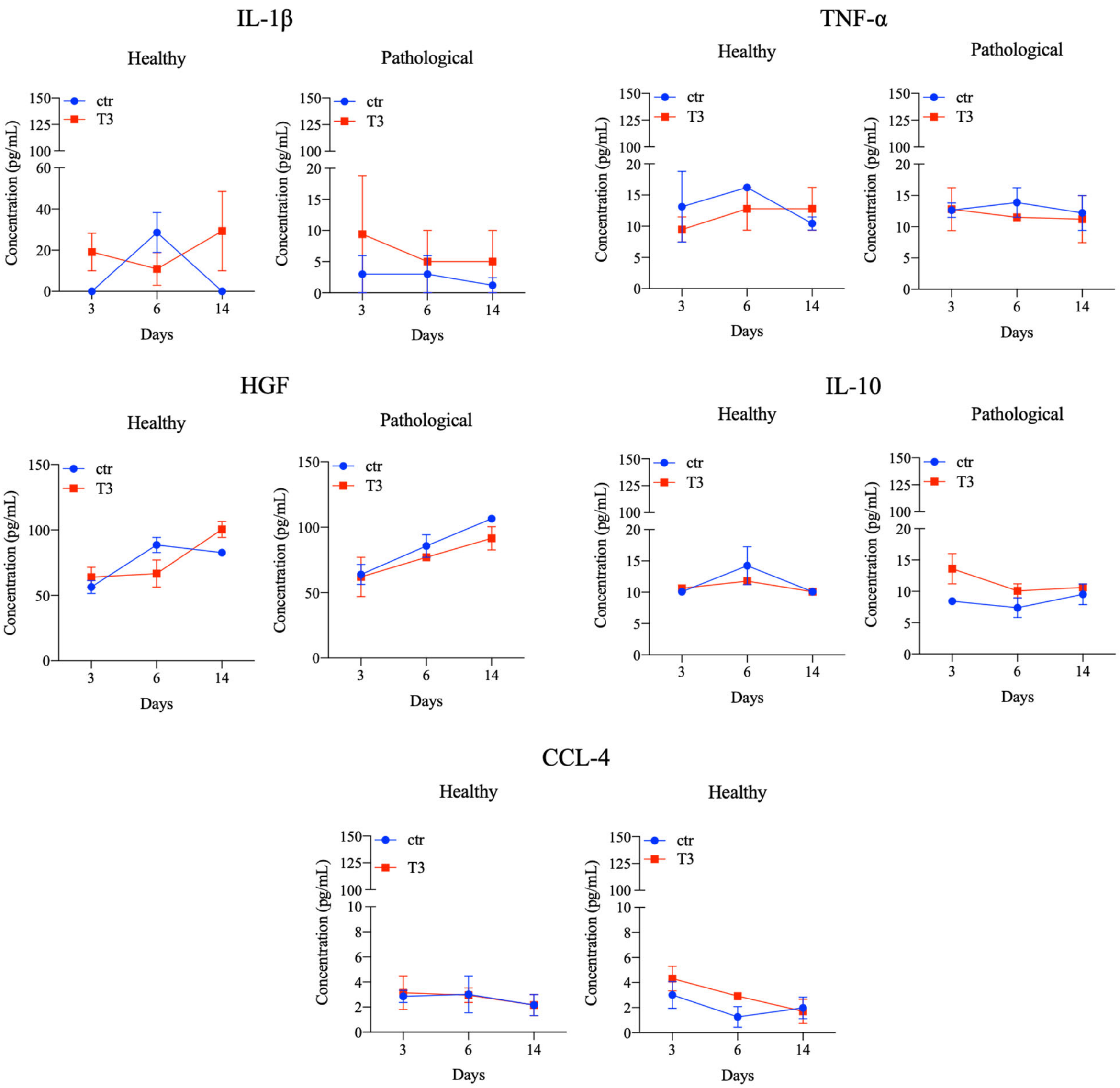

Figure S1. Cytokine profiles of healthy and pathological tissue-derived Tendon Stem/Progenitor Cells (TSPCs) cultured in a medium supplemented with T3 (10-6 M) up to 14 days. Cytokine levels (pg/mL) were measured in culture medium at various time points (3, 6 and 14 days) using a bead-based multiplex immunoassay. Results are shown as mean±SD (N = 2).
